# Review of Issues on Residual Malaria Transmission

**DOI:** 10.1093/infdis/jiab084

**Published:** 2021-04-27

**Authors:** Pierre Carnevale, Sylvie Manguin

**Affiliations:** 1 Institut de Recherche pour le Développement, Portiragnes, France; 2 HydroSciences Montpellier, Institut de Recherche pour le Développement (IRD), CNRS , Université Montpellier, Montpellier, France

**Keywords:** Residual malaria transmission, *Anopheles* vectors, outdoor biting behavior, vector control methods, vector behavior changes

## Abstract

Residual malaria transmission is the actual maintained inoculation of *Plasmodium*, in spite of a well-designed and implemented vector control programs, and is of great concern for malaria elimination. Residual malaria transmission occurs under several possible circumstances, among which the presence of exophilic vector species, such as *Anopheles dirus*, or indoor- and outdoor-biting vectors, such as *Anopheles nili*, or specific behavior, such as feeding on humans indoors, then resting or leaving the house the same night (such as *Anopheles moucheti*) or also changes in behavior induced by insecticides applied inside houses, such as the well-known deterrent effect of permethrin-treated nets or the irritant effect of DDT. The use of insecticides may change the composition of local *Anopheles* populations, such as *A. arabiensis* taking up the place of *A. gambiae* in Senegal*, A. aquasalis* replacing *A. darlingi* in Guyana, or *A. harrisoni* superseding *A. minimus* in Vietnam. The change in behavior, such as biting activity earlier than usually reported—for example, *Anopheles funestus* after a large-scale distribution of long-lasting insecticidal nets—or insecticide resistance, in particular the current spread of pyrethroid resistance, could hamper the efficacy of classic pyrethroid-treated long-lasting insecticidal nets and maintained transmission. These issues must be well documented in every situation to elaborate, implement, monitor, and evaluate tailored vector control programs, keeping in mind that they must be conceived as integrated programs with several well and appropriately coordinated approaches, combining entomological but also parasitological, clinical, and social methods and analyses. A successful integrated vector control program must then be designed to reduce transmission and incidence rates of malaria morbidity and overall mortality.

After a successful period of malaria burden reduction with scaling up of long-lasting insecticidal net (LLIN) distribution, indoor residual spraying (IRS), and artemisin combined therapy, along with intermittent presumptive treatment, rapid detection tests, and possible other approaches [1], malaria remained a worrying public health problem, and its control is at a crossroads [2].

Residual malaria transmission (RMT), which corresponds to *Plasmodium* transmission, could be considered according to 3 main biological components: (1) entomological, such as remaining transmission even after well-done vector control programs; (2) parasitological, corresponding to remaining *Plasmodium* parasites in the blood (or elsewhere in the body, such as in the liver), even after well done and adapted drug administration; and (3) clinical, with remaining clinical illness even after appropriately conducted treatment with correct drugs and dosage. The remaining vectors and parasites could be due to resistance (to insecticide or drugs respectively) and several other biological and socioeconomic factors; in the present article we consider only residual *Plasmodium* transmission, main factors involved, and issues for vector control.

Despite high coverage of insecticide-treated nets (ITN) or IRS, the main vector control methods currently implemented, outdoor and evening or early morning malaria transmission occurs in many malaria-endemic regions and is considered RMT [3]. It represents a key challenge across all malaria-endemic countries. RMT is defined as the transmission of *Plasmodium* that remains once universal coverage of LLINs (>80%) and/or maximal coverage of IRS has been achieved using insecticides to which the local vectors are susceptible [4].

Malaria transmission still occurs in many endemic countries and this could be due to various factors, including insecticide resistance spread, vector behavior and environment changes, and the role of secondary vectors, to cite a few. Actually, vector control methods implemented indoors have the greatest impact on mosquitoes entering, biting, resting, or/and living inside these treated houses, such as endophilic, endophagic, and anthropophilic mosquitoes. Indoor malaria transmission and morbidity rates have been greatly reduced in the last decades with the scaling up of LLINs and IRS [1].

Inversely exophilic, exophagic, zoophilic mosquitoes are not in contact with insecticide-treated surfaces and could maintain some levels of malaria transmission outdoor even in properly treated areas. There is great variability in the behavior (biting and resting) of *Anopheles* between and within species and populations, and outdoor biting could be a natural or an induced behavior due to the insecticide used inside the house.

## NATURAL VARIABILITY OF *ANOPHELES* POPULATIONS

In Africa, South of Sahara, the main vectors, *Anopheles gambiae* and *Anopheles funestus* are essentially anthropophilic, endophilic, endophagic, biting mainly during the second part of the night [5, 6], while *Anopheles arabiensis*, also belonging to the *A. gambiae* complex, has a more plastic behavior, being often quite zoophilic, exophilic, and exophagic. Variability in biting and resting behaviors were noticed with different species [5] in natural conditions without any vector control intervention.

**Figure 1.**
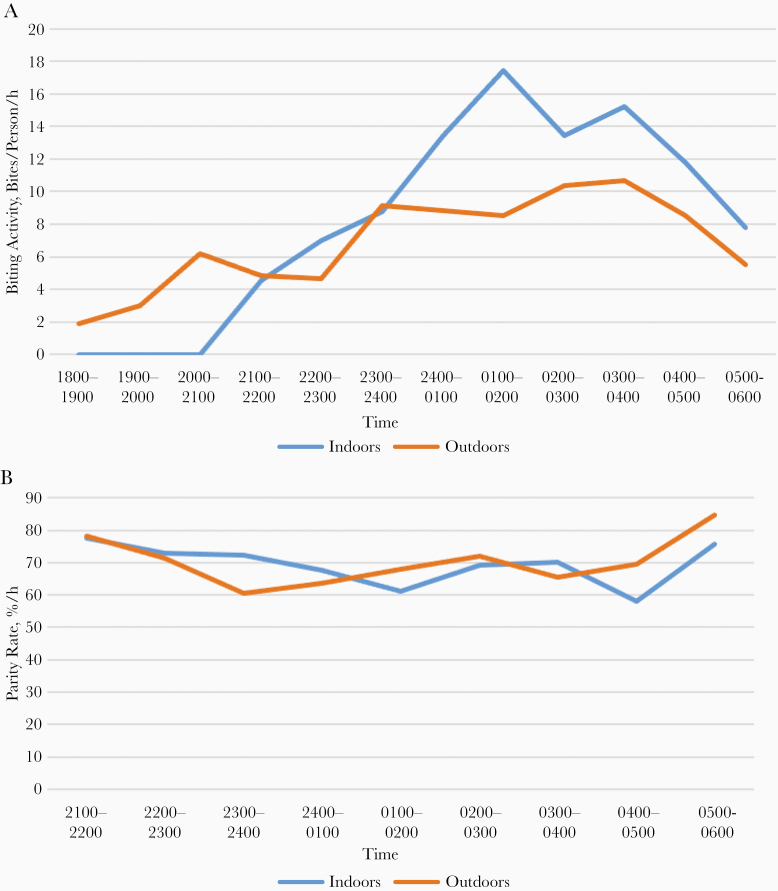
Biting activity and parity rate of *Anopheles nili* for indoor (*blue*) and outdoor (*orange*) human habitations. *A,* Biting activity as hourly number of bites in humans. *B,* Parity rate per hour.

For instance, in Africa south of the Sahara, in rural forested area of Congo (Brazzaville), near permanent rivers such as Lououlou and Louholo, night catches on humans showed that *Anopheles nili* bites outdoors as well as indoors [7] with 3 successive steps: bites began outdoors while people stay outside for usual social activities, then bites indoors and outdoors were similar, while people progressively entered the house for sleeping, an then bites indoors increased at a largely higher rate than outdoors, while most people of the village are sleeping inside their houses (Figure 1A). It clearly showed that the behavior of *A. nili* adapted to human behavior. However, malaria transmission could also occur outside, which was reported when an infected bite was noticed at 2200 outdoors. The *Anopheles* populations biting indoors and outdoors seemed the same, as the parity rate was similar all night (Figure 1B) with an average of 64.8% (n = 895) inside and 67.4% (n = 420) outside.

In Burkina Faso, 12% of the well-known anthropophilic vector species, *A. gambiae,* were attracted to cattle odor traps, and only 40% of specimens caught inside houses had blood fed on humans [8]. In Sao Tome, *A. gambiae* appeared exophagic and fed on dogs [9], and in Bioko Island (Equatorial Guinea), the same species was reported as partially exophagic and biting early at night [10] (after initiation of some vector control programs).

In Eritrea, 36% of infective bites occurred outdoors [11]; in Northeastern Tanzania, 12% of malaria transmission occurred before sleeping time [12]; and in Uganda, up to 36% of indoor and 49% of outdoor transmission occurred before sleeping time [13]. In Latin America, the main malaria vector is *Anopheles darlingi*, greatly anthropophilic, biting during sleeping hours or early in the morning [14], but the main biting peak was also noticed in the evening [15], and outdoor biting was observed in French Guiana [16]. In Nicaragua, in some areas with mainly *Plasmodium vivax*, 50% of infected bites occurred before sleeping time [17].

In Southeast Asia, the level of behavioral heterogeneity of *Anopheles* species and populations according to ecological situations is even higher than on the other continents [18]. *Anopheles dirus* is mostly anthropophilic, exophagic, and exophilic (Table 1), but in Lao People’s Democratic Republic some populations appeared having endophagic and endophilic trends [19]. In western Thailand, populations showed a more zoophilic behavior than anthropophilic with an early biting peak [20]. Other *A. dirus* populations can also blood feed during the daytime in dark forested areas [21]. *Anopheles minimus* can be observed with anthropophilic or zoophilic tendencies, as well as endophilic and endophagic behavior, according to region [18]; the biting behavior of *Anopheles maculatus* (biting earlier than *A. dirus* or *A. minimus*) changes according to locality [3].

**Figure 2.**
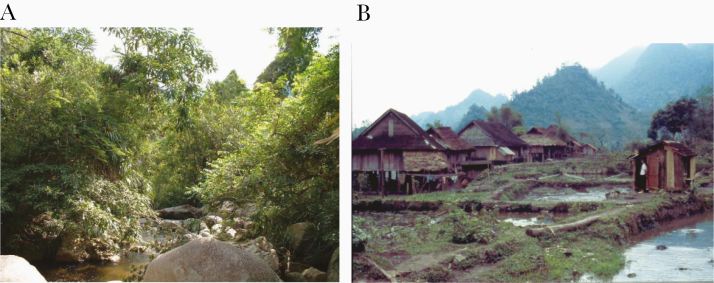
Breeding sites of *Anopheles* vectors in Vietnam. *A, Anopheles dirus* from Khan Hoa Province (photograph provided by P. C.). *B, Anopheles minimus* from Hoa Binh Province (photograph provided by S. M.).

An in-depth study on the adult ecology of *A. minimus* and *A. dirus* was undertaken in the Khanh Hoa Province of Vietnam [22] with a trial on the effects of bed nets impregnated with permethrin. The observation showed that the distribution of *A. dirus* is restricted to forests and areas where the forest has been replaced mainly by plantation of jackfruit, citrus, and rubber (Table 1). Its density was very low during the dry season, with breeding sites restricted to deep forests. *A. minimus* is breeding in streams in the vicinity of the village (Figure 2B) and it appeared strongly endophagic in all seasons, but the highest densities were noticed during the dry season, because during the rainy season the speed of water flow in the larval habitats and small streams, and along river banks, is too fast and larvae are flushed out (Figure 2B). The biting activities of both species occurred indoors and outdoors throughout the night, but inside houses the biting peaks were not the same; *A. dirus* had an earlier peak than *A. minimus,* and *A. dirus* had higher densities outdoors than indoors.

The results of this study showed that the number of specimens collected during biting and resting collections decrease during the second part of the night (after 0200) and that fully fed females were found outdoors on wall surfaces, suggesting that *A. dirus* leaves the houses the same night and fulfills the rest on the gonotrophic cycle outside. Considering that people usually stay outside the nets until 2100, it was estimated that 40% of *A. dirus* and 17% of *A. minimus* bite before 2200 showing thus the potential of RMT even if permethrin-treated nets (PTNs) are implemented in villages. In the same study done in Vietnam, the *A. dirus* biting peak was much higher outdoors than indoors, while for *A. minimus* the human-landing density was 10-fold higher indoors than outdoors. Implementation of PTNs (0.2 g active ingredient/m^2^) induced a reduction of 94% of the indoor biting pressure of these main vectors, even for unprotected people living in the same house who benefited from the mass effect of the nets [22]; this reduction of human-vector contact was most likely due to the deterrent or excito-repellent effect of PTNs.

In another study by Garros et al [23], also done in Khanh Phu, Vietnam, the wide use of PTNs coincided with the significant reduction of the prevalence of *A. minimus* from 100% to 2% between 1999 and 2002, replaced by its sibling species *A. harrisoni*, which prevalence increased from 3% in 1997 to 88% in 2002. Marchand et al [22] concluded that “normal use of treated nets during sleeping hours may be expected to prevent about 80% of the number of bites from *A. minimus*, but due to its early biting habits, at most 60% of those from *A. dirus*, since the seasons of both species alternate, it may be predicted that full use of impregnated nets will, at least initially, have less impact during the rainy season, when *A. dirus* is most important. This effect may be quite marked since, in addition, this species is also more active biting outdoors.” This change in *Anopheles* species composition may have important consequences on RMT and the role of the so-called secondary vectors should not be neglected [24].

## INDUCED CHANGES

**Figure 3.**
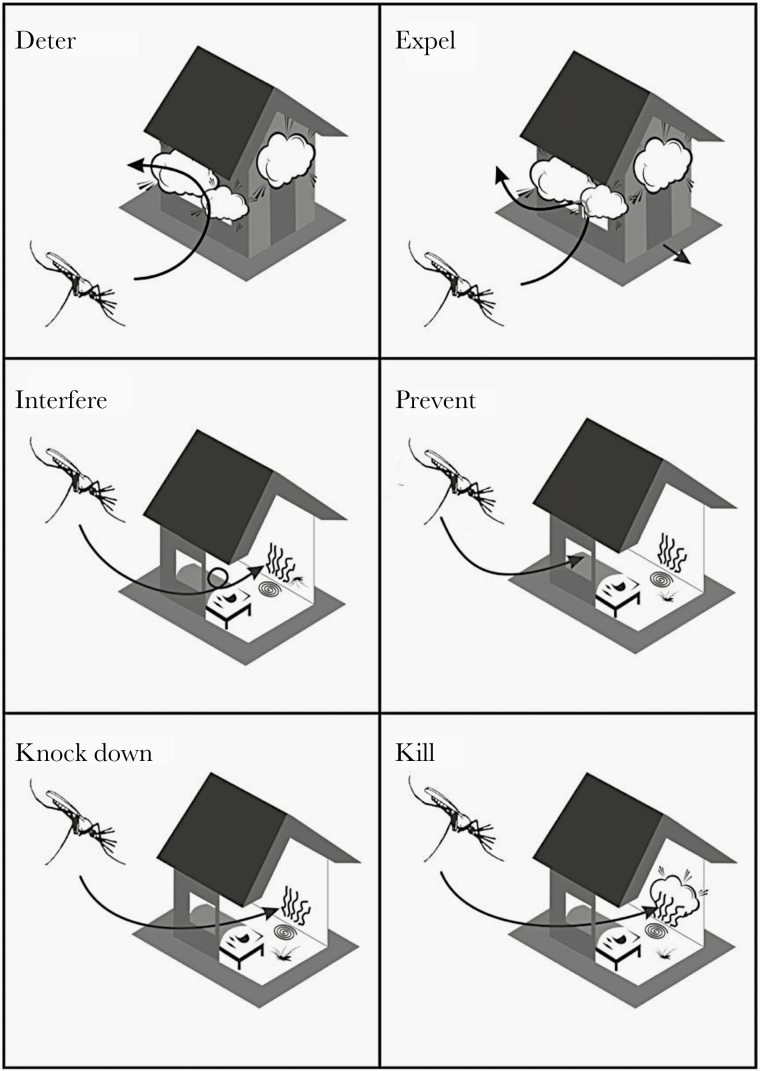
Main behaviors induced by chemicals implemented in houses for vector control (source: Guillaume Carnevale).

Implementation of some insecticides inside a house can have different impact on the behavior of mosquitoes (Figure 3), such as (1) a deterrent effect (avoidance or reduction of mosquitoes to enter inside a treated house); (2) an expellent or repellent effect (mosquito enters the house but, owing to insecticide irritancy, leaves the treated house quickly, before having received enough dose of insecticide); (3) an interference effect on normal flight and biting behavior; (4) a preventive effect (keeping mosquitoes from entering the house); (5) a knock-down effect for mosquitoes coming near the impregnated surface to bite the host; and (6) a killing or insecticide effect of the chemical uses.

For Durnez and Coosemans [3], insecticides could induce 3 main behavioral shifts. The first is behavioral plasticity, which corresponds to mosquitoes having a high degree of irritability or repellency even at the first exposure to insecticide [25], able to detect its presence and then avoid it [26]. This is also considered “protective avoidance” and could be observed in the case of large-scale LLIN or IRS implementation. Endophilic mosquitoes can change to outdoor behavior after enough contact with the insecticide; they can survive, and malaria transmission still occurs. The second behavioral shift is protective behavior, such as exophagy, exophily, zoophily, and early biting, which also leads to a short (if any) contact with the insecticide inside the house. Mosquito species, populations, or subpopulations may present a high or low repellency response [27]. The third shift is behavioral resistance, in which insecticide induces a selection of mutations favoring the mosquito’s survival, such as insecticide resistance. This happens after and with implementation of indoor vector controls.

The impact of indoor vector control operations could lead to some RMT in several ways. These include (1) a shift in biting or resting behavior, from endophagic, endophilic anthropophily to exophagic, exophilic zoophily; (2) a change in species composition within the same complex or group, such as *A. arabiensis* in place of *A. gambiae, Anopheles rivulorum* in place of *A. funestus, or A. harrisoni* in place of *A. minimus* [23]; and (3) increasing importance of local or secondary vectors, such as *Anopheles aquasalis* after elimination of *A. darlingi* in Guyana [28] or exophagic *Anopheles barbirostris* in Thailand [29].

### Shifts in Biting and Resting Behavior

**Figure 4.**
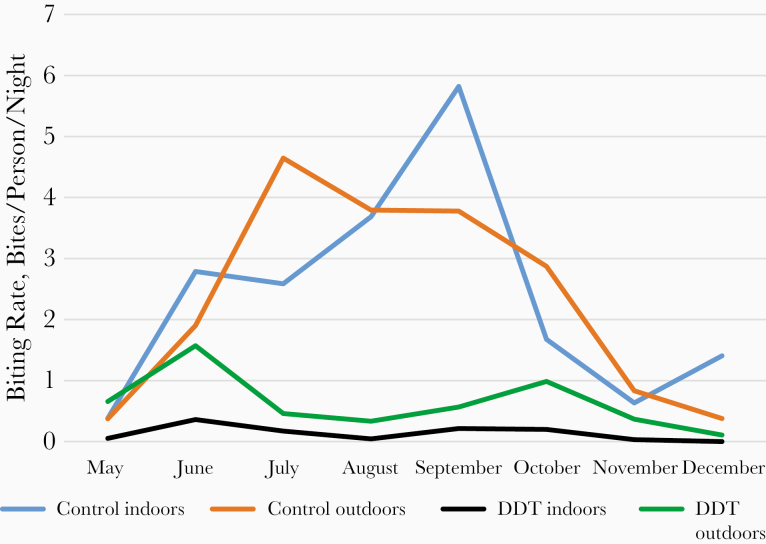
Monthly human biting rate of *Anopheles gambiae* in control and DDT-treated houses of the pilot zone of Bobo-Dioulasso, Burkina Faso [28].

The irritant effect of DDT inducing and increasing the exophilic behavior of *A. gambiae* was well observed in DDT-treated houses of the Pilot Zone of Bobo-Dioulasso (Burkina Faso) [30], where it showed the important following results (Figure 4). In nontreated areas, the biting rates of *A. gambiae* were similar inside and outside (*P* = .93). In DDT-treated areas, the biting rate of *A. gambiae* was significantly higher outside than inside (*P* = .003), clearly showing the irritant effect of the product increasing the exit of mosquitoes; this phenomenon was observed during the 8 months of the trial (Figure 4). In dieldrin-treated villages, the biting rates were similar inside and outside (*P* = .93). Compared with control areas, DDT significantly reduced the biting pressure both inside (*P* < .001) and outside (*P* = .04). The reduction of the biting rate inside DDT-treated houses was of great epidemiological importance for malaria control, while the significant increase in the biting rate outside versus inside DDT-treated houses is of great concern for the pursuit of malaria control, despite the efficient insecticide use of IRS.

**Figure 5.**
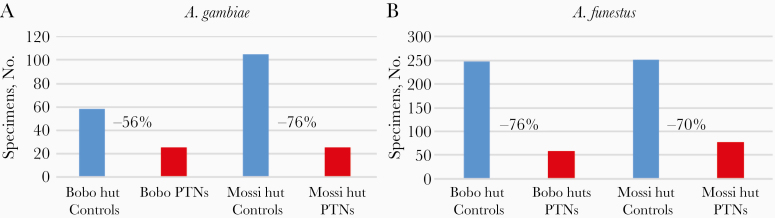
Number of specimens of *Anopheles* vectors caught in controls and permethrin-treated nets (PTNs) in Soumousso experimental huts. *A, Anopheles gambiae*. *B, Anopheles funestus.*

**Figure 6.**
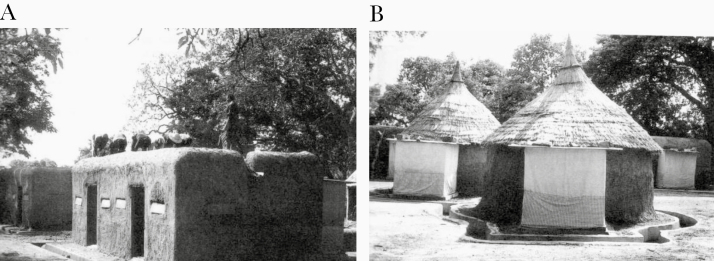
Experimental huts in the World Health Organization (WHO) collaborative field station in Soumousso, Burkina Faso. *A,* Bobo hut design. *B,* Mossi hut design. (Photographs provided by Frédéric Darriet) [29].

The deterrent effect of permethrin was well noticed during the first trial of ITN in the experimental huts of Soumousso, Burkina Faso [31], where samples of *A. gambiae* caught indoors with PTN was reduced by 56% in Bobo huts and 76% in Mossi huts (Figures 5A and 6), compared with huts with untreated nets. For *A. funestus,* the sample size was reduced by 76% in Bobo huts and 70% in Mossi-type huts (Figures 5B and 6).

**Figure 7.**
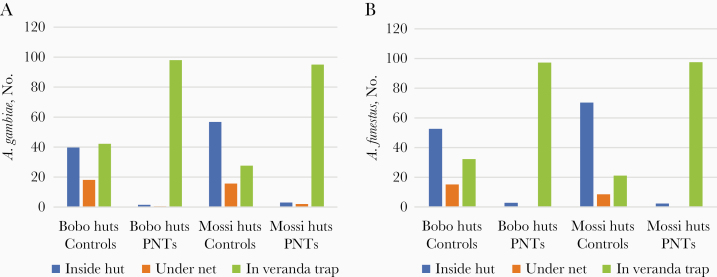
Number of *Anopheles* collected in 3 locations inside the experimental huts, inside the hut (*blue*), under nets (*orange*), or in veranda traps (*green*) in Bobo and Mossi huts with either untreated nets (Controls) or permethrin-treated nets (PTNs). *A, Anopheles gambiae*. *B, Anopheles funestus*.

In addition, locations where specimens were collected during morning hour (eg, inside the hut, the net, and the veranda trap) clearly showed the expel effect of PTNs. The results in Figure 7 clearly showed that most specimens (>95%) were collected inside veranda traps in huts equipped with PTNs for both species, *A. gambiae* (Figure 7A) and *A. funestus* (Figure 7B), which confirmed the expel effect of PTNs, which increases the exit behavior (exophily) of both species.

This increase in exophilic behavior (fewer mosquitoes entering treated huts and more specimens leaving quickly the treated huts) is epidemiologically important, as it clearly shows that PNT confers great personal/familial protection. However, if more mosquitoes are active outside, more will bite and transmit malaria parasites to unprotected people. Therefore, for proper public health management, high (>80%) or universal coverage is absolutely required.

Outdoor RMT has been consistently reported in many areas where interventions such as LLIN or IRS in houses were implemented without much impact [4, 32], while the question of the need for outdoor vector control for malaria elimination was raised [33]. Variations in the ecology of the local vectors, such as shift in biting time from late to early biting [3], increased outdoor biting behavior [34], and changes in species composition [23] have been shown to contribute to maintain transmission [35]. The change in behavior was well noticed in The Gambia [35], as *Anopheles coluzzii,* well known to be anthropophilic, endophilic, endophagic, and a late-night biter [36], and *A. arabiensis,* known to be more zoophilic, exophilic, exophagic, and an early-night biter [3], had similar biting patterns after large-scale distribution of LLINs and IRS.

In Benin, decreased proportions of endophily and endophagy in *A. gambiae* sensu lato (s.l.) populations were observed after IRS and ITN interventions [36]. In the IRS arm, the endophilic rate went from 67.1% before to 4.5% after vector control intervention, whereas in the control arm, the endophilic rate stayed stable at 51.7% (*P* > .05). In the LLIN arm, endophilic rates also decreased after vector control intervention. For the endophagic rate, proportions of mosquitoes biting indoors in the IRS arm decreased from 67.1% before intervention to 42.9% after intervention, while for the LLIN arm, the reduction in endophagy was less pronounced but still significant, going from 71.3% to 57.5% [37].

Similar observations were reported from Tanzania, Benin, Bioko Island (Equatorial Guinea), and Nigeria, where the increasing use of ITN or IRS raised the proportions of outdoor feeding (exophagy) malaria vector populations such as *A. gambiae* and *A. funestus* [32, 38–40]. No shift to increased exophagy of *A. gambiae* after vector control implementation was reported from Tanzania [32, 41], Burkina Faso [42], or The Gambia [43].

### Shift in Biting Time

In Kenya, a shift was observed to an early evening biting time of *A. gambiae* s.l. after implementation of PTN [44]; the same was also reported from Tanzania with *A. gambiae* and *A. funestus* [45, 46]. In Benin, a shift to early morning biting activity was noticed after implementation of a large-scale coverage of LLINs [38].

In Dielmo, Senegal, *A. funestus* showed a behavioral change in biting activity after 2 massive deployments of LLINs, in July 2008 and July 2011. Anthropophilic and endophilic behavior remained, while diurnal feeding was adopted, essentially on humans [47]. Classically, the biting cycle of *A. funestus* presented an indoor peak at 0100–0300 and an outdoor peak at 0200–0500 [48], while in Dielmo increasing aggressiveness was observed between 0700 and 1100, corresponding to the time when people are not under LLINs but are instead involved in early household and farming activities [47].

It is clear that in such circumstances, malaria transmission is still occurring despite LLINs inside houses or indoor IRS, and this situation induces RMT of great concern, because the ways to eliminate outdoor transmission are not quite available. However, shifts in biting behavior have not systematically been noticed with ITN or IRS implementation. In Djoumouna village (Congo), the temporary presence inside a house of a bed net impregnated with deltamethrin (12.5 or 25 mg active ingredient/m^2^) has not induced any shift in the biting cycle of *A. gambiae* [49]. This was also reported from Bioko Island (Equatorial Guinea) [10], Kenya [50] with PTNs, and The Gambia [43] and for *A. funestus* in Kenya [50].

### Shift in Species Composition of the *Anopheles* Population

In several studies, a shift was noticed from indoor to outdoor feeding behavior, which could be due to a shift in species composition, for example, from *A. gambiae* to *A. arabiensis,* which is more zoophilic and exophilic than *A. gambiae* [32, 34, 51, 52]. In Vietnam, a shift from *A. minimus* to *A. harrisoni*, which is more exophagic and exophilic, was reported after the wide use of permethrin-treated bed nets [23].

In Dielmo, Senegal, a shift in species composition in the *A. gambiae* complex after implementation of LLINs nets was described with a drastic decrease in the proportions of *A. coluzzii* and *A. gambiae* after the introduction of LLINs, which was concomitant with an increase in the proportion of *A. arabiensis* [53]. In Kenya and Tanzania, where ITNs were implemented at large scale, the proportion of *A. arabiensis* increased while the densities of *A. gambiae* and *A. funestus* decreased [34, 51].

This phenomenon was also noticed after IRS implementation in Kenya, where *A. funestus* disappeared and was replaced by the more exophagic *A. rivulorum* [54] or *Anopheles parensis* [55], which took over the breeding sites of the declining species. However, the same vector species were noticed after ITN implementation in Bioko Island, Equatorial Guinea [10], or Kenya [50].

Understanding the contribution of outdoor resting *Anopheles* to RMT is important in scaling up vector control toward malaria elimination in South Africa [56]. In KwaZulu-Natal Province (South Africa), the main vectors were *A. funestus* and *A. arabiensis* [57]; the former, highly anthropophilic and endophagic, was well controlled by repeated IRS campaigns done for several years and nearly disappeared [58]. For *A. arabiensis,* which bites and rests outdoors, its control is less amenable to being controlled by indoor vector control operations and is greatly involved in RMT [59].

Other species are also involved in RMT, such as *Anopheles merus* (*A. gambiae* complex) in Mozambique [60] and Mpumalanga Province, South Africa [61]; *Anopheles vandeeni*, an outdoor resting mosquito and member of the *A. funestus* group [62], considered a secondary vector [63]; and *A. parensis*, mainly zoophilic and resting indoors or outdoors, which was found to be positive to *Plasmodium falciparum* circumsporozoite (CSP) [56] and could therefore be involved in RMT in the northern KwaZulu Natal Province of South Africa.

Secondary vectors, such as *A. rivulorum, Anopheles leesoni,* and *A. parensis* were also incriminated in Tanzania [64–66], as well as *A. rivulorum* and *Anopheles longipalpis* in Kenya [67, 68], all of which may contribute to RMT. However, the global lack of studies on secondary vectors prevent any precise information on their specific role in malaria transmission [24].

### Shift in Biting Preference

Large-scale use of ITN in Kenya induced a shift of *A. gambiae* and *A. funestus* from humans (protected) to animals, mainly cattle [69]; the same shift from human, not readily accessible, to locally available animals was also noticed in Burkina Faso [8]. However, a large number of studies found no particular trophic deviation induced by ITN or IRS on *A. gambiae* [39, 41, 42, 70], *A. funestus* [41], or *A. arabiensis* [71].

### Shift in Resting Place

In French Guiana *A. darlingi* disappeared from inside houses after IRS campaigns, while staying outside in the peridomestic environment [16]. Shifts to exophily were observed in species from all geographic regions, including Afrotropical (*A. arabiensis*), Australasian (*Anopheles farauti*), Oriental (*A. dirus*), and Neotropical (*A. darlingi*) [3].

## VECTOR CONTROL METHODS FOR OUTDOOR-BITING MOSQUITOES

Among the available vector control methods currently used, most of them target indoor-biting (endophagic) and indoor-resting (endophilic) mosquitoes. Very few approaches are efficiently designed to control exophagic or exophilic mosquitoes, although new approaches have recently been developed. This is a review on some currently available methods to control outdoor-biting *Anopheles* vectors.

### LLIN Implementation

**Figure 8.**
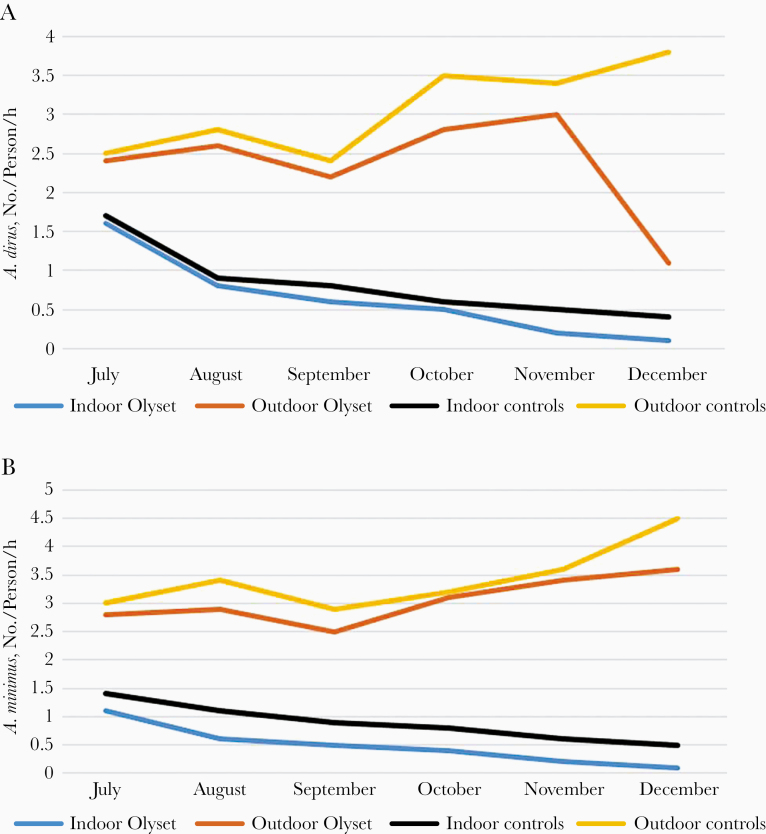
Monthly evolution of the biting density (bites per person per hour) of *Anopheles* vectors indoors and outdoors in controls and Olyset net villages. A, *Anopheles dirus*; B, *Anopheles minimus*.

In the Rattanakiri Province of Cambodia, a trial of Olyset LLINs (Permethrin) was implemented against *A. dirus* and *A. minimus* (Yeng Chhelang and Lek Sandy, unpublished report). For *A. dirus*, the average densities of bites per person per hour were 0.6 and 0.8 indoors in Olyset and control villages, respectively, and 2.4 versus 3.1 outdoors, owing to the exophagic behavior of this species (Figure 8A). The bite densities (bites per person per hour) were significantly higher outdoors than indoors in control (*P* = .002), as well as in Olyset villages (*P* = .04), confirming the common exophagic behavior of this species (Figure 8A).

When comparing Olyset villages and controls, densities were similar inside (*P* = .43), as well as outside (*P* = .13). According to Chhelang and Sandy (unpublished report), the total indoor biting density, per person per hour, was reduced by 71.4% in Olyset areas and by 55.6% in control areas. Outdoors, the reduction rate was only 14.3% in Olyset areas, and the density increased by 16.1% in control villages, showing that even against an exophagic vector such as *A. dirus*, implementation of PTN could still be worthwhile in reducing human-biting pressure.

For *A. minimus*, a reduction rate of 72.2% in indoor density was reported, compared with 30.8% in control areas, while there was a reduction rate of 3.2% in outdoor density in Olyset areas and an increase of 11.8% in control areas (Figure 8B). Trends of the monthly evolution of bite density (bites per person per hour) were remarkably similar. Hourly bite densities were significantly higher outdoors than indoors in both Olyset (*P* = .002) and control areas (*P* = .002), and similar inside (*P* = .80) and outside (*P* = .25) treated and control areas (Figure 8B).

The entomological data recorded after the implementation of Olyset nets not only clearly showed a reduction in indoor biting densities, but it was also very efficient in decreasing the whole population of exophilic mosquitoes by 71.4% for *A. dirus* and 72.2% for *A. minimus* (Figure 8). Another important result to underline is that during the 7-month period, the number of malaria cases was reduced by 91.1% and deaths by 100% (Chhelang and Sandy, unpublished report).

### Larval Source Management

#### Fish-Based Biological Control

**Figure 9.**
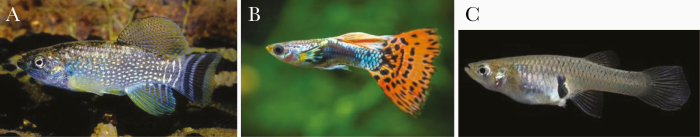
Fish used in biological control against mosquito larvae. *A, Aphanius díspar*. *B, Poecilia reticulata* (guppy). *C, Gambusia affinis.*

In Djibouti, when the main malaria vector was *A. arabiensis,* a large-scale larval control program was implemented in the capital based on the autochthonous larvivorous fish *Aphanius dispar* (Figure 9A), along with the use of locally *Bacillus thuringiensis* or *Bacillus sphaericus* [72]. First, all *A. arabiensis* breeding sites were localized, and 800 wells appeared as actual or potential sites. Besides, the local larvivorous fish *A. dispar* was collected and reared. Then, each well received 5–10 fish, which were checked for their presence regularly and restocked if needed. Local or temporary ponds with stagnant rain water were also treated first with temephos (0.1–0.5 ppm) and then with *B. thuringiensis* (0.1–0.2 g/m^2^) or *B. sphaericus* (1–5 g/m^2^), the latter product lasting longer than *B. thuringiensis* (3 weeks). This large larval control program induced a 73% reduction of positive breeding sites, and 90% of expected clinical malaria cases were avoided [72].

*A. dispar* was also successfully used in Assab, Ethiopia, against *Anopheles culicifacies adanensis* [73]. Cisterns, wells, and barrels found positive for larvae received this fish with monthly restocking, which was enough to maintain actual control. It thus appeared that 34% (range of 18%–60%) of unstocked site were positive, while 1.6% only of the fish-treated breeding sites still harbored some larvae. It is important to underline that stocking of larvivorous fish in wells and household water storage containers was well accepted by the participants, and, based on the results of this study, larvivorous fishes were introduced on an operational scale for the control of malaria transmission in Assab, with voluntary participation of the population.

A study on *A. dispar* in India [74] showed that the mean (standard deviation) daily consumption of larvae in laboratory was as follows: *Anopheles stephensi,* 128 (0.2) to 204 (6); *Culex quinquefasciatus* 24 (4) to 58 (10); and *Aedes aegypti,* 43 (5) to 68 (2). In water tanks, the fish *A. dispar* reduced larval counts by 93% by day 7 and by 98% by day 21 (*P* < .01), showing high larval propensity (Figure 9A).

According to these studies from Djibouti and India, and considering that *A. stephensi* is now present in Djibouti [75], larval control based on *A. dispar* should be implemented to control this efficient urban malaria vector. It was recently reported that *A. stephensi* invaded Djibouti and Ethiopia, potentially spreading to other areas of Africa [76]. This invasion of *A. stephensi* increases the major threat of urban malaria transmission in East Africa and requires urgent control measures to prevent malaria epidemics in cities, which would cause a public health disaster [77].

**Figure 10.**
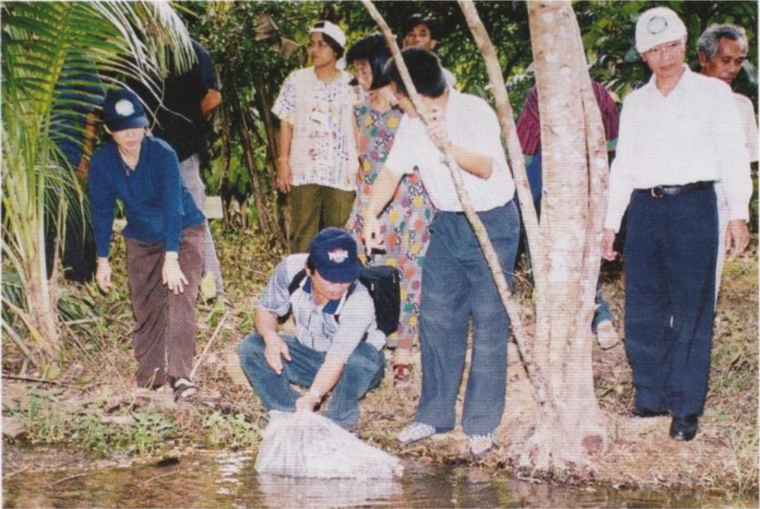
Releasing larvivorous fish, Chanthaburi, Thailand (photograph by Tawat Kantasri, courtesy of Frédérick Gay) [78].

In Thailand, other species of fish, including guppy and *Gambusia* (Figures 9B, 9C, and 10) were used for the control of vectors with community participation [78, 79], in addition to IRS with deltamethrin and ITN. Environmental modifications to reduce the larval habitats were also implemented, targeting *A. minimus* and *A. maculatus,* but it seemed unfeasible for *A. dirus,* despite the fact that it is the most potent vector.

#### Larviciding, Environmental Management, and Outdoor Residual Spraying

In KwaZulu Natal Province (South Africa), *A. funestus* almost disappeared after repeated IRS campaigns, leaving room for *A. arabiensis,* which became the main vector, primarily responsible for the bulk of RMT in South Africa. *A. parensis,* while being known as strongly zoophilic, was also found to be positive for *P. falciparum* CSP [56]. As Burke et al [54] mentioned, the 2 *P. falciparum*–positive specimens of *A. parensis* were caught resting outdoors, which highlights the importance of addressing RMT in South Africa by targeting both indoor- and outdoor-resting vectors. Therefore, besides continuing IRS programs, intensive larval source management programs, including winter larviciding and community outreach programs designed to educate on personal protection measures and treatment seeking, are under development to address this issue.

**Figure 11.**
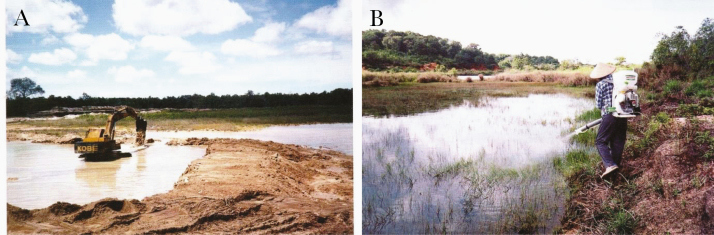
Mosquito larval source reduction in Indonesia. *A,* Elimination of manmade breeding places carried out by the Department of Public Works. *B,* Larviciding by health department personnel (photographs by Bangkit Hutajulu, courtesy of Frédérick Gay) [80].

In Indonesia, interesting intersectoral collaborations were developed, for instance, one with the Ministry of Agriculture that provided a legal base regarding regulations on irrigation and planting schedules, which proved critical to malaria vector management success in Java. The Ministry of Public Works has strived to reduce the sources of larval habitats in endemic areas, and vector control was done with environmental management (Figure 11A), larviciding spray (Figure 11B) and community participation, as well as classic IRS [80].

**Figure 12.**
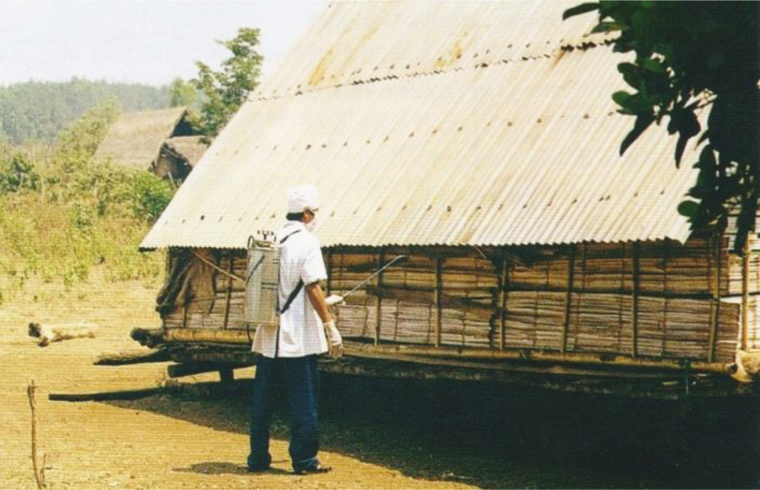
Outdoor residual spraying at a malaria epidemic focus in Dak Lak Province, Vietnam (photograph by Huu Thuy, courtesy of Frédérick Gay); [81].

To control an outbreak in Central Vietnam, larval source reduction and outdoor residual spraying were implemented (Figure 12). The outdoor residual spraying control method deserves further study and evaluation of its impact in reducing RMT [81].

#### Long-Lasting Insecticide-Treated Hammocks

**Figure 13.**
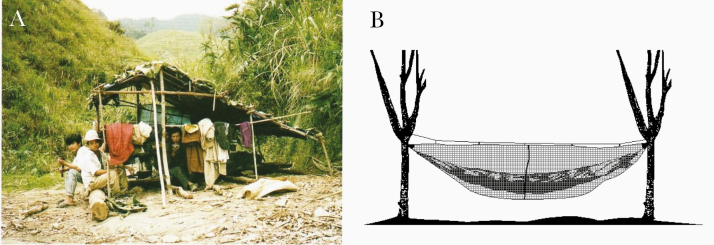
Field conditions and protection against outdoor-biting mosquitoes. *A,* Woodcutters in their temporary shelter in Vietnam (photograph by Vo Hinh, courtesy of Frédérick Gay) [82]. *B,* Long-lasting insecticide-treated hammock (source: Guillaume Carnevale).

A main issue in malaria control concern migrants farmers, woodcutters who are living in temporary shelters without any protection (Figure 13A) [82]. One solution could be to provide them with LLINs or/and long-lasting insecticide-treated hammocks (LLIHs) (Figure 13B) when they go to work in forested areas prone to *A. dirus* [83–86]. Another way to prevent them from malaria transmission would be to use topical repellent or/and impregnated clothes to reduce the biting pressure.

A trial using LLIHs with the classic LLIN Olyset Net sewn at the backside of the hammock was implemented in Western Cambodia in Pang Rolim village, *A. minimus* being the main vector, and Dey Krahorn Leu village, with *A. dirus* predominant [83]. Implementation of treated hammocks reduced by 45% *A. minimus* bites in both villages, while for *A. dirus* a reduction of 46% was observed only during the second survey (*P* = .005). These results mean that the personal protective effect of LLIHs against exophagic vectors and nuisance due to mosquitoes was variable according to species, villages, and surveys. Nearly half of the *A. minimus* bites could be avoided by using LLIHs. A similar result was obtained against *A. dirus* but only during the second survey (end of the rainy season), and no evidence of protection was found in the middle of the rainy season [77].

In central Vietnam, an interesting biological and social study was recently implemented in Khanh Hoa Province considering vectors and common use of impregnated nets (ITNs or LLINs) according to place of residence (village) and/or temporary work (farm, forest) [87]. ITN/LLIN coverage in the village was reported to be >90%. The vast majority (>90%) of individuals owned a forest farm plot; 72% sometimes slept in the farm huts overnight, and 33.2% sometimes stayed overnight in the forest. Only 12.1% of forest workers regularly used a net overnight in the forest, and 1.1% sometimes did so. Mosquitoes were collected on a single cow bait tent trap outside, as well as human-landing catch indoors and outdoors. The results confirmed the absence of *A. dirus* in villages with universal coverage of treated nets, and the importance of this coverage inside and outside farm huts and in forests [81].

The normal sleeping time in the community in farm huts was at 2100, while 45% of the biting peak of *A. dirus* s.l. and 100% of *A. maculatus* s.l. occurred before 2100. For *Anopheles* mosquitoes outdoor in the forest and inside farm huts, the peak biting activity was highest at 2000–2100. Three *A. dirus* s.l. were found to be sporozoite positive, 2 with *P. falciparum*, both collected outdoors, 1 near the farm hut and 1 in the forest, and 1 with *P. vivax,* also outdoors at farm huts.

This study in Vietnam showed entomological inoculation rates for *P. falciparum* of 17.8 infectious bites per person per year in the outdoor farm hut site and 25.3 per person per year in the forest, specifically from *A*. *dirus* s.l. The entomological inoculation rate for *P. vivax* from *A. dirus* s.l. in the forest site was also 25.3 infectious bites per person per year [87]. These observations are well in line with some others done in Central Vietnam, showing that human outdoor activities, especially at night, favor exposure to malaria vectors that cannot be prevented by sleeping under LLINs and some risk factors relating to evening outdoor exposure may have been missed in previous studies [88].

#### Insecticide-Treated Plastic Sheeting

**Figure 14.**
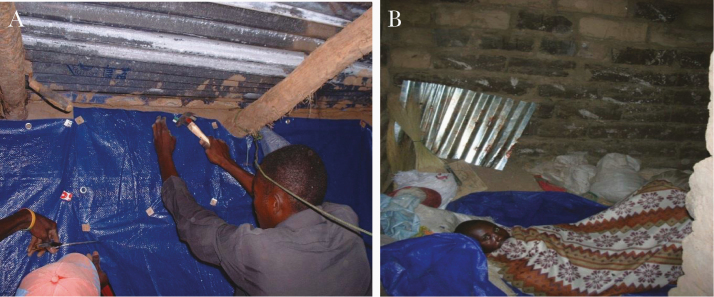
Use of ZeroFly insecticide-treated plastic sheeting (ITPS) in villages in Angola. *A,* Hanging ITPS on the wall. *B,* Sleeping on ITPS spread on the floor. (Photographs provided by P. C.)

Considering that biting rates in farm huts were comparable to those seen in the forest, because farm huts offered little to no protection due to poor structure, freely allowing entry of mosquitoes through the walls and floors, it is interesting to try here the recently developed insecticide-treated plastic sheeting (ITPS). ITPS can be pinned on walls, inside or outside huts, by people themselves, and on the floor, serving several purposes (Figure 14).

ITPS was successfully used in refugee camps in Sierra Leone [89] and Afghanistan [90], and ZeroFly ITPS was also implemented in India [91, 92] against *A. stephensi* and *A. culicifacies.* Entomological and parasitological data showed the high efficacy of ZeroFly sheeting in controlling malaria. It is worth noting that, for Sharma et al [92], the introduction of ZeroFly plastic sheeting in a community-based intervention program was an operationally feasible way to contain malaria, especially in high-transmission areas.

A village-scale control program around Balombo town (Benguela Province, Angola) was implemented in 2007 using deltamethrin-treated ITPS as a wall lining or ZeroFly [93], used either alone or combined with deltamethrin-treated LLINs or after λ-cyhalothrin IRS. The results showed an overall reduction of 90% of the entomological inoculation rate by *A. funestus* and *A. gambiae* and 80% of the *Anopheles* density. In addition, 70% reduction in *P. falciparum* prevalence, 65% reduction in the gametocyte index, and 55% reduction in the number of cases in children <9 years old were also recorded [93].

**Figure 15.**
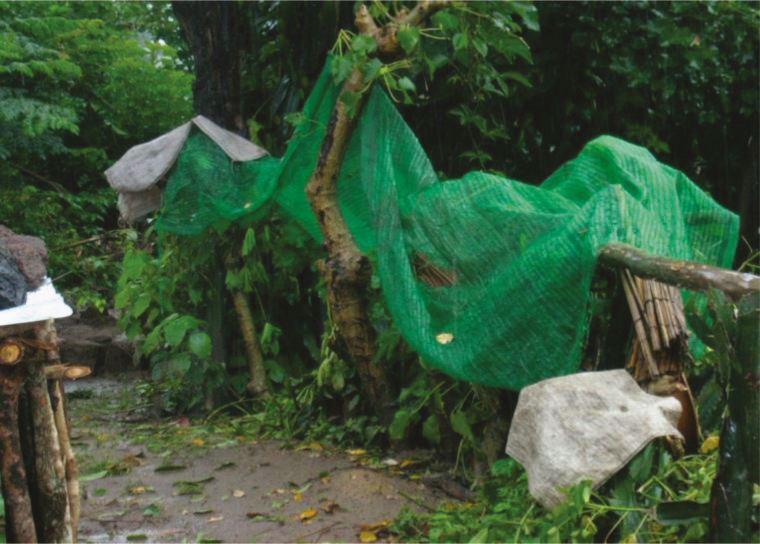
Example of a wall lining withdrawn from houses and laid on the fence in Angola (photograph provided by P. C.).

One main concern was the withdrawal of ITPS by the population in some villages (Figure 15), often discarded outside, underscoring the need for information education communication campaigns. Therefore, ITPS deserves special attention as it can also be used on the outside walls of huts and temporary shelters of farmer migrants in Southeast Asia to protect them against outdoor *Anopheles* vectors when staying near their houses for usual social events before sleeping.

It should also be possible, if and where feasible, to place ITPS between well-known breeding sites, such as streams for *A. minimus* (Figure 2) or pools for *A. dirus*, and villages to form a barrier to protect people. Hand-made fences around villages to protect against wild animals could also be used to support ITPS, which would provide a protection to villagers against outdoor-biting mosquitoes (Figure 15).

#### Zooprophylaxis

A recent analysis of 34 articles dealing with blood feeding and resting habits of *A. arabiensis* considered 4 types of zooprophylaxis: passive, active, combination, or insecticide zooprophylaxis [94], defined as follows. Passive zooprophylaxis is the natural prophylactic effect of cattle seen when cattle density within a community is increased. Active zooprophylaxis refers to the deliberate introduction of domestic animals to divert mosquitoes away from human settlements toward other nontransmitting hosts. Combination zooprophylaxis is the use of ITNs and IRS integrated with livestock placed in a separate shed, to induce a push-pull effect, thereby reducing disease incidence. Finally, insecticide zooprophylaxis is the treatment of cattle by sponging or dipping the cattle with insecticides to pass on a lethal dose to blood-feeding mosquitoes. For Asale et al [94], the studies examined showed that zooprophylaxis can have positive, negative, or no effects on malaria vector control.

Two studies analyzed the push-pull combined actions with the deliberate introduction of LLINs and IRS used as the pushing factor, while zoophilic and opportunistic mosquito species such as *A. arabiensis* are attracted by domestic animals (ie, the pulling factor) [95, 96]. Other studies used the additive role of LLINs in zooprophylaxis, which demonstrated by modeling that scaling up mass coverage of LLINs to 80% in the community and ensuring 80% coverage of livestock treatment with pyrethroids could lead to a global reduction and elimination of the disease [97, 98].

According to a recent review of literature, zooprophylaxis could be part of an effective strategy of integrated vector management to reduce malaria transmission under specific ecological and geographic conditions [99]. However, the current scientific evidence base is inconclusive on the role of socioeconomic factors, the optimal distance between livestock and human sleeping quarters, and the effect of animal species and densities on zooprophylaxis, because 2 opposite phenomena could occur, zooprophylaxis or zoopotentiation. The first, zooprophylaxis, corresponds to the reduced malaria risk in areas where predominant mosquito species do not prefer human hosts, where livestock are kept at a distance from human sleeping quarters at night, and where mosquito nets or other protective measures are used. While the second, zoopotentiation, refers to the fact livestock may actually draw mosquitoes to humans, increasing malaria transmission, which occurs where livestock are housed within or near human sleeping quarters at night and where mosquito species prefer human hosts [99]. In fact, various parameters interfere starting with the *Anopheles* species being targeted, and each situation has to be carefully studied before embarking in a large-scale zooprophylaxis program, but the possibility has to still be kept in mind [100].

#### Reproduction and Swarming

Another innovative approach is targeting male mosquito mating behavior for malaria control [101], based on sound knowledge of the reproduction of the concerned *Anopheles* species, such as *A. arabiensis* [102, 103], a vector mainly involved in RMT following the effect of LLINs in decreasing *A. gambiae* and *A. funestus*. For a long time, a large amount of work was devoted to the sterile insect technique preferentially targeting males [104–106], and it is gaining new emphasis with RMT not yet controlled by currently available techniques such as IRS and LLINs [107–109]. Considering the well-known risk of reinvasion by mosquitoes, the mass release of sterile males can be done only in remote villages or islands, as developed for the control of *Aedes albopictus* in La Réunion Island [110].

### Personal Protection With Skin Repellents or Permethrin-Impregnated Clothes

A number of studies has been devoted to skin repellents [102–106, 111–115], with several recommendations of use, such as the those provided by the former French organization Afssaps [116]. Special attention should be given to the use of skin repellents for newborns, babies, and small children. The French Pediatric Society [106] stated that before infants are old enough to walk on their own, skin repellents must be avoided unless there is a high risk of vector-borne diseases, such as an outbreak of dengue or other arboviruses, but infants must be protected by treated mosquito nets; for those aged 6–12 months, recommendations include once-daily para-menthane-3,8-diol or PMD (Citriodiol) 20%–30%, N,N-diethyl-m-toluamide (DEET) 10%–30%, or IR 35/35 20% [106].

Other recommendations and considerations include conditions and limits of use, duration, mosquito species of the area, perspiration, use of sunscreens [117], time to spend in at-risk areas and during at-risk periods [118]. Skin repellents confer protection against outdoor-biting vectors and deserve further attention for their promotion as a public health tool.

Soap containing 20% DEET and permethrin (either 0.5 or 1.0%) were tested on Penang Island, Malaysia against *Anopheles lesteri* and other outdoor mosquito species [119]. An interesting efficacy was noticed, with 80%–100% reduction in mosquito landing and biting rates according to the species tested and residual effects were registered up to 4 hours. In high mosquito densities, small percentages of *A. lesteri* landed on treated skin. The use of the soap repellent formulations could be envisaged for personal or familial protection, while considering cost-effectiveness, safety, sustainability, and acceptability. Such soap could also be used to “wash and treat” clothes; this could be tested in terms of efficacy and efficiency against outdoor mosquitoes to protect day and night workers exposed to biting pressure.

Permethrin-impregnated clothes were largely tested and used against several species of mosquitoes in various situations [120–126]. Clothes could be treated by dipping, as with nets, the method used in several parts of Vietnam, by spraying [127], or already industrially treated [128]. Treated clothes can keep their efficacy for 2 months and resist to 6–8 washes (with cold water) when normally used. Resistance could be increased to 33 washes (cold water) with an increased dose of permethrin; the efficacy is quickly reduced when the clothing is washed in hot water (60°C) with detergent. Clothes could also be treated with repellents such as DEET [127] but with short residual activity.

Permethrin-treated clothes could also be used with skin repellents on “unprotected” area of the body [129], and such combinations showed great efficacy against exceptionally aggressive *Culiseta impatiens* (2400 bites per hour). Permethrin on clothes gave a 93.4% protection, DEET on skin gave 99.7% protection, and using both conferred 99.9% protection [130].

A field evaluation of personal protection methods against outdoor-biting mosquitoes was conducted in 2 study sites in Xieng-Ngeun district, Luang Prabang Province, northern Lao People’s Democratic Republic, where the more abundant species were *Culex vishnui* s.l. and *A. albopictus,* and the putative malaria vectors were *Anopheles barbumbrosus* s.l., *A. minimus* s.l., *A. barbirostris* s.l., *A. dirus* s.l., *A. maculatus* s.l., *Anopheles epiroticus,* and *Anopheles umbrosus* [126].

The study showed that: (1) mosquito coils in a metal casing worn on a belt provided 92.3% protection against all mosquito species during the afternoon (1200–1800) and 68.8% during the evening (1700–2300); (2) the combination of permethrin-treated clothing, plus PMD or para-menthane-3,8-diol (menthoglycol or Citriodiol) resulted in 68.2% protection in the afternoon and 52.3% in the evening; (3) the combination of untreated overalls with PMD resulted in 55% protective efficacy, and only 25.2% in the evening; and (4) long permethrin-treated clothing resulted in 61.1% protection during the afternoon and 43% in the evening.

The conclusion of this study was that portable mosquito coils were highly protective against outdoor-biting mosquitoes, although there are safety concerns related to their use. The combination of permethrin-treated clothing and PMD repellent represent an alternative treatment for protection against outdoor-biting mosquitoes.

The use of synthetic repellents against mosquitoes (Table 2), particularly DEET, has raised some issues on safety and health risks to humans and the environment. Therefore, plants-based repellents should be increasingly studied to serve as safer alternatives to synthetic repellents. In addition the raw material to produce plant-based mosquito repellents can be readily accessible, locally accepted, and even quite popular in some communities, and affordable to low-income communities, while quite efficient in preventing mosquito bites. At present, the development of natural product-based repellents with more effective and long-lasting protection is under study. Many active ingredients have been studied, most of them being essential oils [131–134]. Studies have shown that 2 plant-based compounds such as β-caryophyllene oxide [135] and vetiver oil [136] have great potentials as natural insect repellents against *A. minimus, A. dirus*, *A. albopictus,* and *A. aegypti*. These plant-based repellents were shown to be environmentally friendly and safe for public use [135, 137].

**Table 2.** Active Ingredients of Skin Repellents and Concentrations by Age Group and for Pregnant Woman^a^

Age Group	Active Ingredients	Concentration, %
30 mo to 12 y	PMD (Citriodiol)^b^	20–50
	IR 35/35	20–35
	DEET^b^	20–35
	KBR 3023^c^	20–30
>12 y	PMD (Citriodiol)b	20–50
	IR 35/35	20–35
	DEETb	20–50
	KBR 3023c	20–30
Pregnant women	IR 35/35	20–35

Abbreviations: IR, insect repellent; DEET, N,N-diethyl-m-toluamide; KBR 3023, icaridine or picaridine.

^a^Data from Sorge et al [106].

^b^Not used in patients with a history of seizure.

^c^Not used for >1 month.

#### Attractive Toxic Sugar Bait

The attractive toxic sugar bait method is based on the “lure and kill” strategy, in which the instinct of mosquitoes to search and feed on sugar sources is exploited [138, 139]. The attractive toxic sugar bait is coformulated with low-risk toxic substances, such as boric acid, and can be deployed in bait stations or sprayed on plants, providing an interesting way to control outdoor mosquitoes [140–146].

#### Nanosynthetized Insecticides

A paint, containing 2 organophosphates, chlorpyrifos and diazinon, and insect growth regulator, pyriproxyfen, was tested under laboratory conditions for 12 months after World Health Organization pesticide evaluation scheme (WHOPES) phase I procedures [147]. After treatment, delayed mosquito mortality rates were high (87%–100%), even in organophosphate-resistant *A. gambiae* females on all surfaces except cement treated at 1 kg/12 m^2^. One year after treatment, delayed mortality rates were 93%–100% in organophosphate-resistant females on nonporous surfaces at both doses. On cement, death rates were low 12 months after treatment regardless of the dose and the resistance status. Fecundity, fertility and adult emergence were reduced after treatment, even at the lower dose (*P* < .01). A reduction in fecundity was still observed 9 months after treatment at both doses (*P* < .01), and adult emergence was reduced at the higher dose (*P* < .01) [148]. Owing to its spatial mortality pattern (mortality at distance without contact with insecticide treated surface) [149], insecticide paint for an outdoor residual spraying could be an interesting way to control outdoor mosquitoes when surfaces of the huts or shelters allow its use.

## Conclusions

The burden of malaria dropped strikingly in the last decade with the large-scale implementation of vector control methods essentially based on LLIN and IRS, and with availability of artemisin combined therapy [1]. However, control programs have to deal with some key problems, such as insecticide resistance [150] and RMT, transmission that is maintained even after well-done vector control operations against susceptible vectors. RMT is due to several factors, such as potential shifts in vector behavior from endophagy to exophagy, from endophily to exophily, and from anthropophily to zoophily. RMT is also occurring owing to the selection of outdoor-biting, resting, or zoophilic behavior of *Anopheles* vector populations (*A. arabiensis*, *A. minimus*) or outdoor-biter species, such as *A. dirus*, the jungle mosquito present in remote forested areas of Southeast Asia, where it presents a serious challenge to malaria control [151]. Change in vector composition is also another factor affecting RMT, with some species taking over breeding sites and replacing the eliminated ones, such as *A. rivulorum* [54], *A. parensis* [55] or *A. harrisoni* [23].

These situations could be quite different according to local conditions and natural or induced changes, with a dynamic process, such as environmental management or modifications, plantations (eg, rice and rubber), deforestation for agricultural projects, social and cultural habits, economical situations, migrants (to and from forested areas), resettled populations, climatic changes, *Anopheles* genetic background, and issues with species identification in complexes or groups such as *A. gambiae*, *A. funestus*, *A. minimus, A. maculatus*, *A. dirus*, to cite a few, in which sibling species cannot be morphologically differentiated [152, 153].

One species can have different behavior and vector capacity according to the region or country: for example *Anopheles aconitus* is a swamp breeder in Indonesia [154], where it is one of the common species. It rests outdoors in bushes, predominantly zoophilic but bites humans readily [155], can enter houses and cattle sheds, and starts biting as early as 1800 until 0100. It can also rest inside houses or cattle sheds, and it is a secondary vector in Orissa (India) but reported as an important vector in Java and Sumatra (Indonesia), even with low vector competence [156, 157].

Therefore, there is no single solution, no magic bullet applicable everywhere, but rather adapted and tailored measures, which can be implemented only after sound knowledge of every situation with comprehensive understanding on biological, social, and economic factors involved in RMT, needing multisectorial coordination to elaborate and implement relevant plan of action, ranging from community-based involvement to international cooperation to prevent reintroduction of malaria at international borders.

Several tools are now available for vector control of outdoor *Anopheles* mosquitoes, and each has its advantages and issues. These tools must be used in an integrated vector management led by field-oriented pluridisciplinary teams targeting the reduction of malaria transmission, morbidity, and mortality rates and the elimination of the disease in the foreseeable future [158].

**Table 1.** Mosquito Taxa and Biting Rate by Setting and Collection Method^a^

Setting	Collection Method	*Anopheles* Taxon	Biting Rate, Bites/Person/Night
Village	Cow bait	*A. maculatus*	0.5
	OHLC	*A. maculatus*	0.1
Farm hut	IHLC	*A. dirus*	4.3
		*A. maculatus*	0.1
	OHLC	*A. dirus*	5.7
		*A. maculatus*	0.5
Forest	OHLC	*A. dirus*	4.5
		*A. maculatus*	0.2

Abbreviations: IHLC, indoor human landing collection; OHLC, outdoor human landing collection.

^a^Data from Hung et al [81].

## Notes

***Financial support.*** This work study was supported by the French National Research Institute for Sustainable Development (IRD).

***Supplement sponsorship***. The supplement is sponsored by TDR, the Special Programme for Research and Training in Tropical Diseases, based at the World Health Organization.

***Potential conflicts of interest.*** Both authors: No reported conflicts. Both authors have submitted the ICMJS Form for Disclosure of Potential of Interest. Conflicts that the editors consider relevant to the content of the manuscript have been disclosed.
